# Primary Hemostasis Disorders as a Cause of Heavy Menstrual Bleeding in Women of Reproductive Age

**DOI:** 10.3390/jcm12175702

**Published:** 2023-09-01

**Authors:** Athanasios Kontogiannis, Alkis Matsas, Serena Valsami, Maria Effrosyni Livanou, Theodoros Panoskaltsis, Panagiotis Christopoulos

**Affiliations:** 1 Second Department of Obstetrics and Gynecology, “Aretaieion” Hospital, Faculty of Medicine, National and Kapodistrian University of Athens, 115 28 Athens, Greece; 2 Hematology Laboratory-Blood Bank, Aretaieion Hospital, National and Kapodistrian University of Athens, 115 28 Athens, Greece

**Keywords:** abnormal uterine bleeding, heavy menstrual bleeding, menorrhagia, primary hemostasis disorders, bleeding disorders

## Abstract

Heavy menstrual bleeding (HMB) is a common clinical condition affecting adolescent and adult women and compromising their quality of life. Primary hemostasis disorders, affecting platelet plug formation, can be the underlying cause of HMB. They comprise a heterogeneous group of diseases with Von Willebrand disease (VWD) being the most commonly diagnosed; other disorders in this group that have been linked to HMB include (a) Glanzmann thrombasthenia, (b) Bernard–Soulier syndrome, (c) Hermansky–Pudlak syndrome, (d) immune thrombocytopenia (ITP), and (e) Ehlers–Danlos syndromes (EDS) and hypermobility spectrum disorders (HSD). Diagnosing these diseases can be challenging, as the basic laboratory investigations can be within the normal range. Thus, identification of specific clinical features and a thorough hematologic workup can be very important, providing the correct diagnosis. Proper diagnosis of the underlying disorder is important, as management may vary accordingly. Although disease-specific management guidelines exist for some of these disorders such as VWD and ITP, due to the rarity of most primary hemostasis disorders, the best approach for the management of HMB in these women remains elusive. The goal of this study was to create an informative, comprehensive review of the primary hemostasis disorders that have been linked to HMB. This study provides a summary of the basic published information regarding epidemiology, pathophysiology, clinical phenotype, diagnosis, and treatment of HMB in those diseases and serves as a reference guide for further reading.

## 1. Introduction

**Figure 1:**
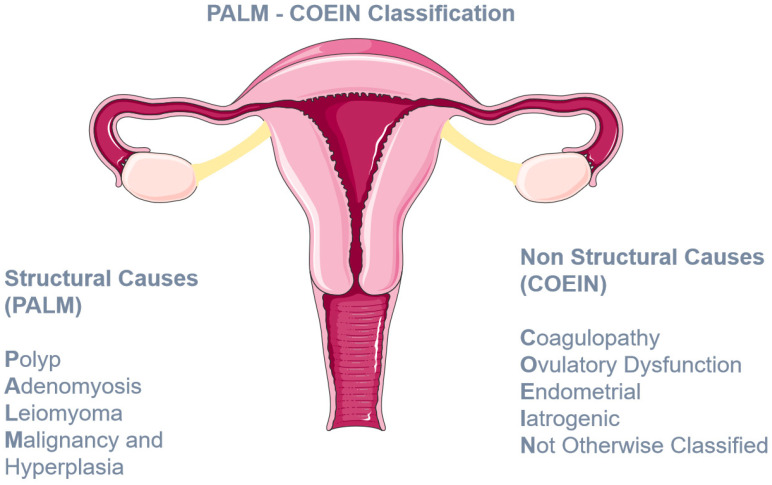
The PALM COEIN classification system of AUB.

Abnormal uterine bleeding (AUB) is a common debilitating condition for women of reproductive age, having a significant impact on their physical and mental health [1]. It is estimated that 10–62% of adolescents with heavy menstrual bleeding (HMB) may have an underlying inherited bleeding disorder—coagulopathy [2]—which may lead to repetitive doctor visits, long investigations, and several hospitalizations [3]. Furthermore, it comes with a high financial burden for the healthcare system [4]. Abnormal uterine bleeding is the term used to denote the disturbed menstrual bleeding manifesting as either excess menstrual blood loss or abnormal menstrual timing. Following the 2009 FIGO World Congress in Cape Town, abnormal uterine bleeding replaced the old and confusing term dysfunctional uterine bleeding. Heavy menstrual bleeding is the subcategory of AUB characterized by increased menstrual bleeding and/or prolonged bleeding (over 7 days). It has been proposed to replace the outdated term “menorrhagia”, although sometimes the two terms are used interchangeably in the current literature [5]. In this review, we have also used the term menorrhagia in several instances in order to reflect the terminology used in the original published articles. The causes of AUB are classified according to the PALM COEIN System of Nomenclature (as indicated in Figure 1). Each letter of the acronym represents a broad category of disorders, namely polyp; adenomyosis; leiomyoma; malignancy and hyperplasia; coagulopathy; ovulatory dysfunction; endometrial; iatrogenic; and not yet classified [5]. In this review, we will focus on disorders of coagulation-hemostasis, and specifically those affecting primary hemostasis. Hemostasis involves three steps: primary hemostasis or formation of a platelet plug, secondary hemostasis, or coagulation and fibrinolysis. Primary hemostasis occurs promptly after endothelial injury. Local vasoconstriction is followed by activation of Von Willebrand factor (VWF), which binds to exposed collagen proteins. Specifically, VWF binds GPIb receptors on the platelet membrane and so it acts as a bridge for the connection of platelets to the subendothelium. Finally, the adhesion of platelets to the subendothelium is facilitated by the synergistic activation of receptors GPIIb/IIIa and collagen receptors GPVI and α2β1. The firm platelet aggregate ensures resistance to the high shear forces of blood flow.

Further platelet aggregation is accomplished by release of adenosine 5′-diphosphate (ADP), serotonin and thromboxane A2 (TxA2), the latter being synthesized from arachidonic acid formed by platelet membrane phospholipids. 

The final primary hemostatic step is a change in platelet morphology, which now expresses various phospholipids in their membrane surface, necessary for coagulation factor assembly and initiation of secondary hemostasis [6]. As explained above, primary hemostasis is a complex process involving multiple steps and mediators and several disorders can lead to its impairment, with the end result of dysfunctional platelet plug formation and an increased hemorrhagic risk. Primary hemostasis disorders can be divided into (1) vascular disorders (affecting the endothelium), such as hereditary hemorrhagic telangiectasia, disorders of the connective tissue (including Ehlers–Danlos Syndromes and osteogenesis imperfecta), and small vessel vasculitis; (2) Von Willebrand disease; (3) platelet function disorders, including Bernard–Soulier syndrome, Glanzmann thrombasthenia and Hermansky–Pudlak syndrome; and (4) thrombocytopenias. Both males and females may be affected by these disorders, as in the majority of cases they follow an autosomal inheritance pattern [7,8,9,10,11]. However, women with disorders of primary hemostasis are more likely to experience symptoms than their male counterparts due to the additional hemostatic challenges of menstruation and parturition. In this review, we focus on the primary hemostasis disorders that have been associated with abnormal uterine bleeding in women of reproductive age; Von Willebrand disease, Glanzmann thrombasthenia, Bernard–Soulier syndrome, Hermansky–Pudlak syndrome, immune thrombocytopenia, Ehlers–Danlos syndromes (EDS) and hypermobility spectrum disorders (HSD). For each of these disorders, we briefly review the epidemiology, pathophysiology, clinical picture, prevalence of abnormal uterine bleeding, diagnosis, and management, including the symptom-specific management of HMB. Our aim is to create an informative, comprehensive review for physicians who are most likely to first encounter women with HMB—especially general practitioners, obstetricians, and gynecologists—with the aim of raising awareness for an often-overlooked problem with a high socioeconomic impact [4]. 

## 2. Primary Hemostasis Disorders

### 2.1. Von Willebrand Disease

Von Willebrand disease (VWD) is the most common inherited bleeding disorder, with an estimated prevalence of 0.6–1.3% [12], and it usually manifests as mucosal bleeding. Affected women are more likely to experience symptoms of bleeding than men, due to the additional gynecologic-specific hemostatic processes. It accounts for 5–24% of cases of abnormal uterine bleeding in women of reproductive age [13]. Accordingly, it is estimated that 50–92% of women with Von Willebrand disease experience symptoms of AUB. [14].

#### 2.1.1. Pathophysiology

VWD is caused by decreased activity or function of the Von Willebrand factor, secondary to either a quantitative or qualitative defect. VWF is a multimeric glycoprotein which is synthesized and stored in endothelial cells and megakaryocytes and cleared by macrophages in the liver and spleen. It plays a critical role in primary and secondary hemostasis; thus, defective activity can lead to bleeding manifestations. In primary hemostasis, following endothelial damage, VWF binds to exposed extracellular matrix proteins of the endothelial cells and mediates adhesion of platelets, which bind VWF via GPIb receptor. In secondary hemostasis, VWF acts as a chaperone to factor VIII (FVIII), thus preventing premature clearance and degradation [15,16,17].

Historically, in the middle of the 20th century, VWD had been characterized as “pseudo-hemophilia” due to the low FVIII coagulation activity (FVIII:C) which was a cause of diagnostic confusion between hemophilia and VWD. VWF antigen testing became available through immunoprecipitation in the mid-1970s, thus making the diagnosis possible [18,19].

#### 2.1.2. Classification

VWD is subdivided into three categories, according to the International Society on Thrombosis and Hemostasis (ISTH) classification. Type 1 and Type 3 are quantitative defects, with Type 1 characterized by partial deficiency of VWF and Type 3 by absolute deficiency. Type 1C is a subcategory of Type 1 in which the partial deficiency of VWF is due to increased clearance. Type 2 on the contrary, is a group of qualitative defects comprising of the following subgroups: Type 2A which is due to a defect in multimerization, and types 2B, 2N and 2M caused by abnormal ligand binding; increased binding to GPIb receptor, defective binding to FVIII and defective binding with normal multimers, accordingly [8,15].

#### 2.1.3. Inheritance Pattern

VWD follows an autosomal inheritance pattern with low penetrance and variable expressivity. Types 2B and 2M, as well as the majority of type 1 and 2A cases, are autosomal dominant. Type 3 and Type 2N subtypes on the other hand, exhibit an autosomal recessive inheritance pattern. A single case of uniparental disomy has been reported in type 3 VWD [20]. The relative frequency of each subtype is estimated to be 70–80% for Type 1 VWD, 20% for Type 2 and <5% for Type 3 [8,21]. Interestingly, these frequencies are very different when only patients with the severe VWD phenotype are taken into account. Whilst in the overall VWD patient population the frequency of quantitative defects is higher, in the severe VW disease subpopulation, qualitative defects are more prevalent. For example, in a cohort study of 1167 people from 670 families, studied in a hematology reference center in France, the corresponding prevalences were 25% Type 1, 8% Type 3, 66% Type 2 (2A: 18%, 2B: 17%, 2M: 19%, 2N: 12%), and 1% undetermined type [22].

#### 2.1.4. Clinical Manifestations and Epidemiology of HMB

The clinical picture of patients with VWD varies according to the level of residual VWF activity, the category of disease, as well as age and sex. In adults, the most common symptoms are hematomas, menorrhagia, epistaxis, and bleeding from minor wounds. The majority of patients (60 to 80%) experience bleeding following surgery or dental extractions. A well-recognized, potentially life-threatening situation for these patients is gastrointestinal bleeding from angiodysplasia, more often in older patients with qualitative VWF defects. Intraarticular hemorrhage may be the first symptom in patients with type 2N or type 3 VWD. HMB is the most commonly reported symptom among women of reproductive age with VWD. Some of the published research regarding the prevalence of HMB among women with HMB can be found in Table 1. Other than menorrhagia, reproductive tract symptoms include bleeding of an ovarian cyst, and it should be included in the differential diagnosis of women with VWD presenting with abdominal pain [17].

#### 2.1.5. Diagnosis

Standardized bleeding assessment tools (BATs) can be used to identify specific bleeding phenotypes; however, they have certain restrictions, and it is currently suggested that their use should be limited to patients with low suspicion of VWD and that high-suspicion patients should proceed directly to a laboratory evaluation. The recommendation for the general population is that VWD testing should be reserved for patients with personal or family history of bleeding [28,29].

Initial evaluation of women suspected to have VWD includes detailed medical and bleeding history and physical examination. A popular test to screen for women who should undergo further laboratory test was developed by Kouides et al. and includes the following [30].

- Heavy menstrual bleeding since menarche.
- One of the following conditions:
  ■ Postpartum hemorrhage,
  ■ Surgery-related bleeding, and
  ■ Bleeding associated with dental work.
- Two or more of the following conditions:
  ■ Epistaxis, one to two times per month,
  ■ Frequent gum bleeding, and
  ■ Family history of bleeding symptoms.

Women with a positive test result should undergo further laboratory testing. The diagnosis of Von Willebrand disease is based on measurements of Von Willebrand factor antigen, the level of Von Willebrand factor-dependent platelet adhesion, and the coagulant activity of factor VIII. Regarding, the VWF-dependent platelet adhesion, the 2021 guidelines by the American Society of Hematology (ASH), the International Society on Thrombosis and Hemostasis (ISTH), the National Hemophilia Foundation (NHF), and the World Federation of Hemophilia (WFH), suggest that newer assays measuring the platelet-binding activity of VWF (e.g., VWF:GPIbM, VWF:GPIbR) should be preferred over the VWF ristocetin cofactor assay (VWF:RCo) for the diagnosis of VWD. However, this suggestion is based on low-certainty evidence [28]. The diagnosis of qualitative or mixed VWD (Types 2 and 3) is relatively straightforward in comparison to Type 1 disease. The phenotypic characteristic of Type 1 VWD is a partial reduction in the level of intrinsically normal VWF, but determining the optimal cut-off value is challenging and a subject of long-standing debate [17]. To set the diagnosis of Type 1 VWD, the ASH, ISTH, NHG, WGH panel strongly recommends a cut-off value of VWF level <0.30 IU/mL regardless of bleeding, and a VWF level of <0.50 IU/mL for symptomatic patients with abnormal bleeding. Further details regarding the proposed diagnostic assays and specific cut-off values for the diagnosis of each subtype of the disease can be found in the Guidelines [28]. 

#### 2.1.6. Treatment Options for HMB

Therapeutic and prophylactic management of patients with VWD is a very challenging topic which is subject to long-standing and ongoing research. As it is outside of the scope of this research to provide a comprehensive review of this topic, we will focus on the management of heavy menstrual bleeding in these patients. Considering that the prevalence of symptomatic VWD is estimated to be around 1 in 1000 (making it a rare diagnosis) it is difficult to conduct large randomized controlled trials with a sufficient number of participants. Brignardello-Petersen et al., in a summary of three Systematic Reviews addressing the first-line management of menstrual bleeding in women with VWD, which included published research up to October 2019, managed to identify only 1 randomized clinical trial (RCT), 1 observational study and 10 case series, which met their eligibility criteria, and the evidence provided by them was assessed as very low to moderate. Furthermore, the RCT included women with heavy menstrual bleeding due to other hemostatic abnormalities of, and not only VWD. Up-to-date, there is no high-quality evidence to guide the management of women with AUB and VWD. Very recently, the results of a phase 3 randomized controlled clinical trial by Ragni et al., comparing the efficacy of Tranexamic acid (TxA) and recombinant VWF for the treatment of HMB were published. A summary of the evidence provided by the research of Brignardello-Petersen et al., regarding the use of the four most commonly used therapeutic interventions for treating HMB; desmopressin (DDAVP), TxA and hormonal therapy including Oral Contraceptive therapy and Levonorgestrel-Releasing Intrauterine System (LNG-IUS) [29], along with the phase 3 clinical trial by Regni et al., is provided in Table 2.

Although the aforementioned studies provide limited and moderate- to low-quality evidence regarding the relative efficacy of the recommended treatments for treating HMB in women with VWF, to our knowledge, there are currently no published results of high-quality comparative research to guide treatment. Furthermore, the 2021 ASH Recommendations are in line with the results of the, suggesting either hormonal therapy—Combined Hormonal Contraceptives or LNG-IUS—or tranexamic acid over desmopressin for women with VWD and HMB who do not wish to conceive, and tranexamic acid over desmopressin for those who wish to [34]. The results of the VWDMin study by Ragni et al. are unlikely to change these recommendations, since recombinant VWF was not found superior to tranexamic acid in controlling HMB.

### 2.2. Glanzmann Thrombasthenia

Glanzmann thrombasthenia (GT) is a rare genetic bleeding disorder, inherited via an autosomal recessive pattern. Although the exact number of GT cases is unknown, the prevalence is estimated to be 1 in 1,000,000 people. Studies suggest that affected women seem to slightly prevail against men (58% vs. 42%). GT patients can be found all around the world; however, consanguinity seems to be a cause of higher prevalence in populations such as Iraqi Jews, some Arab populations, and French Gypsies [35]. In Iran, GT has been recorded in as many as 1 in 200,000 people [36].

#### 2.2.1. Pathophysiology

The disease is caused by a defect in the platelet membrane glycoprotein IIb/IIIa, also called integrin αIIbβ3 (ITG aIIβ3). This glycoprotein acts as a large heterodimeric cell transmembrane receptor with the major function of mediating platelet aggregation and firm adhesion of platelets to the subendothelial matrix. Mutations of its genes lead to a quantitative (type 1 and type 2) or qualitative (type 3) deficiency in platelet membrane glycoprotein (GP)IIb/IIIa resulting in impaired platelet aggregation. The platelet plug formation is hindered and thus a bleeding tendency occurs [35].

#### 2.2.2. Clinical Manifestations

As it exhibits an autosomal recessive pattern of inheritance, bleeding manifestations in GT seem to be limited to homozygous patients, with the heterozygotes being mostly asymptomatic. Bleeding diathesis manifests soon after birth, although some cases of GT are diagnosed later in life [7]. The phenotype seems to be quite heterogeneous and unpredictable, although purpura in areas of pressure or minor trauma, epistaxis, gingival hemorrhage, and HMB are nearly constant features [37]. Epistaxis seems to be the most common symptom in some studies and is the most common cause of severe bleeding in GT [36,37,38,39]. Epistaxis is typically more severe in childhood. The bleeding tendency of GT is thought to decrease with age; however, this could be an impression because epistaxis is generally more common in children than to adults, leading to a greater chance of inducing a severe episode in those ages [37]. Serious complications can be caused by gastrointestinal bleeding and hematuria, although they are rarer clinical manifestations [7]. Spontaneous, unprovoked bleeding is uncommon in Glanzmann thrombasthenia, as a primary hemostasis disorder. Severe bleeding complications can be encountered during pregnancy and delivery [37]. Although bleeding phenotype varies in severity and frequency, GT is generally considered a severe primary hemostatic disease, as most patients have a history of red blood cell (RBC) or platelet transfusions [40]. Nevertheless, the mortality is low, and the prognosis is excellent with careful supportive care [7]. 

#### 2.2.3. Epidemiology of HMB

All the studies published up-to-date agree on the high frequency of HMB in women and girls with GT. However, the exact number varies significantly among them. Early reports by George et al. recorded a prevalence of 98.2% among female patients (54/55), while the GTR, a prospective observational, international registry that included patients from 15 countries worldwide, estimated a prevalence of 73.6% (67/91) among post-puberal women [37,38,39]. However, a systematic review by Marieke C. Punt et al. that analyzed 4 cohort studies including 154 GT female patients, estimated the percentage of women with HMB to be 22,1% (34/154), significantly different from previous reports; yet still frequent [41]. HMB can be especially dangerous at menarche due to the prolonged anovulatory cycles that increase the bleeding risk during the first periods. Anemia is a common complication of those patients and transfusions are quite often needed during those first episodes [37].

#### 2.2.4. Diagnosis

The full blood count (FBC) usually shows no abnormalities, with the platelet count being at the lower end of the normal range. Prothrombin time (PT) and activated partial thromboplastin time (aPTT), as well as the blood film are also usually normal in GT. However, the bleeding time is prolonged. The LTA (light transmission aggregometry) for GT is characteristic, with platelet aggregation failing to occur with all agonists other than ristocetin (ADP, collagen, thrombin and arachidonic acid). Flow cytometry confirms the defect in the number or function of GPIIb/III receptors. Type 1 is indicated by absence of GPIIb/IIIa, type 2 by reduction in number and in type 3 GPIIb/IIIa may be expressed but is not functional. The platelet function assay (PFA) is prolonged among patients with GT. Finally, genetic analysis confirms the mutations accountable for the disease [35,42].

#### 2.2.5. Treatment Options

General measures for the treatment and prevention of bleeding episodes in patients with GT include the following:

(1) Preventative measures, such as abstinence from bleeding triggers (such as NSAIDS);
(2) Topical measures, such as packing in case of epistaxis;
(3) Antifibrinolytics, mainly used in surgeries and HMB;
(4) DDAVP;
(5) Recombinant Factor VIIa (rFVIIa);
(6) Female hormones for gynecologic complications;
(7) Surgical interventions (such as dilatation and curettage);
(8) Red Blood Cell transfusions;
(9) Platelet transfusions [43]. Platelet transfusions are considered standard treatment during surgeries or trauma and are often used in nonsurgical episodes [43,44]. However, platelet alloimmunization as well as blood borne infection transmission should be taken into consideration every time this therapeutic intervention is considered [45].

#### 2.2.6. Treatment Options for HMB

There is no consensus about the treatment of HMB in females with GT due to the rarity of the disease. Most of our knowledge derives from case reports and case series. As mentioned above, HMB is particularly common in menarche and severe enough to usually require transfusions. When it comes to managing episodes of HMB, besides administering blood products, hemostasis is usually achieved via intravenous infusion of high-dose conjugated estrogen for 1–2 days followed by high doses of oral combined estrogen and progestin, after which combined oral contraceptives (COCs) often need to be used for 2–3 months [46]. After the first post-menarchal ages, antifibrinolytics are generally used as a first line treatment and if they fail to decrease blood loss, oral contraceptives can be useful in eliminating menses [43]. Other hormonal methods that have been used with success in controlling refractory cases of HMB in GT patients include:

(A) Depo-medroxyprogesterone acetate (DMPA) [43,47],
(B) HPO axis suppression (leuprolide and goserelin) [44,48], and
(C) LNG-IUS [49].

The rFVIIa has also been used successfully for this cause [48,50]. When hormonal methods have all failed, surgical interventions including endometrial ablation or endometrial tamponade are the next step [46,51]. Many of the cases mentioned in the literature concern adolescents during their first (or close to their first) menstrual period and are hard to manage. Iron supplementation is also often required. The next Table (Table 3) refers to the treatment methods used in 18 female patients with GT and HMB gathered in the systematic review by Marieke C. Punt et al. based on case reports and case series. No DDAVP was used in GT patients with HMB, and no deaths were recorded [41].

### 2.3. Bernard–Soulier Syndrome

Bernard–Soulier syndrome (BSS) is a rare platelet membrane receptor defect. According to studies from North America, Europe and Japan, the prevalence of the disease has been estimated as less than 1 in 1,000,000 people, although misdiagnosing and underreporting might lead to an underestimation of the prevalence. The syndrome is usually transmitted in an autosomal recessive pattern with only rare exceptions of autosomal dominant forms [9]. In families with consanguinity, the prevalence can be higher [42].

#### 2.3.1. Pathophysiology

The bleeding tendency characterizing BSS patients is attributed to mutations of the Gp1b-IX-V gene complex. This membrane protein complex facilitates two major platelet functions (a) binding of platelets to VWF and thus adhesion to the subendothelial space and (b) the ability of thrombin at low concentrations to activate platelets. When those functions are hindered, due to a dysfunctional complex, clot formation is hampered and thus a bleeding diathesis occurs, which can often be severe. Furthermore, the complex is thought to play a vital role in the process of normal platelet generation in the bone marrow, as indicated by the low platelet count and pathologically big size of platelets in Bernald Soulier patients [9,42].

#### 2.3.2. Clinical Manifestations

The bleeding tendency of BSS patients is usually apparent during the first years of life. Common clinical manifestations include (a) recurrent epistaxis, which is usually the most common cause of severe bleeding, (b) gingival bleeding and (c) trauma-induced hemorrhage. However, there are great variations in the clinical profile among BSS patients, as well as in the severity of the symptoms which can worsen or become alleviated after childhood. Pregnancy can pose severe risks on BSS patients or progress uneventfully [9,42]. Some patients experience gastrointestinal hemorrhage and hematuria. Joint bleeding and spontaneous intracerebral hemorrhage have only rarely been reported. Bernard–Soulier syndrome is often diagnosed during childhood following early manifestations. However, cases of adulthood diagnoses have been described, mostly in mild phenotypes and in some autosomal dominant forms [42].

#### 2.3.3. Epidemiology of HMB

Heavy menstrual bleeding can be an important clinical manifestation in the course of the disease. According to a systematic review by Marieke C. Punt et al. that included data from 4 cohort studies, overall, 25% of women with Bernard–Soulier syndrome (13/52) have a present or past history of HMB [41]. In a tertiary care center in India, out of 1100 cases of HMB, 104 of which had no pelvic pathology or hormonal disorders, 1% (1/104) had BSS (0.09% of the total) [52].

#### 2.3.4. Diagnosis

When a hemostatic disease is suspected, the FBC reveals in most cases a moderate to mild thrombocytopenia which is disproportionate to the bleeding symptoms, thus aiming to differentiate it from immune thrombocytopenia. The initial diagnostic evaluation also includes a blood film examination that can show giant platelets. The bleeding time is prolonged but aPTT and PT are normal. In a person with clinical bleeding manifestations, the combination of those facts can predispose the specialist to the potential diagnosis of BSS. It is important to note though that platelets in BSS patients can be larger than the automatic counter cut-off and therefore in order to avoid underestimation of their number, an optical/manual method is preferred [42,53]. Platelet aggregation in LTA is absent only in response to ristocetin (and normal in response to ADP, collagen, and arachidonic acid), but unlike VWD this defect cannot be corrected by the addition of normal plasma. Flow cytometric analysis confirms the diagnosis by demonstrating the decrease in the affected membrane receptor [53]. Finally, it is possible to identify the genetic abnormality by molecular genetics [42].

#### 2.3.5. Treatment Options for HMB

Treatment in general in BSS shares a lot of similarities with GT and follows the general rules mentioned in the GT section [43]. In particular, for HMB in BSS patients, there is no standard therapy since data are limited. Treatment is based upon general management recommendations for HMB as mentioned in GT patients. It is recommended that those people should be managed by specialists and multidisciplinary teams to ensure optimum treatment [54]. The 2020 systematic review mentioned above included 14 BSS patients from case reports and case series and their therapeutic interventions are summarized on Table 4. No deaths from HMB were recorded [41].

### 2.4. Hermansky–Pudlak Syndrome

Hermansky–Pudlak syndrome (HPS) is a congenital qualitative platelet disorder, characterized by platelets with low amounts of dense granules, making it a delta granule storage pool disorder. It is transmitted via an autosomal recessive pattern and has an estimated worldwide prevalence of about 1 in 100,000 to 1 in 1,000,000, although much more common in Northwest Puerto Rico and the Swiss Valois, where the prevalence reaches 1 in 1800 people [10,55].

#### 2.4.1. Pathophysiology

To date, there have been recognized at least 10 different genes responsible for this genetically heterogenous syndrome. Mutations in HPS1 gene are the most frequently reported. Those genes encode proteins responsible for synthesis or function of lysosome-related organelles, such as melanosomes and platelet dense granules. The aftermath of their genetic mutations is the disruption in production or trafficking of those organelles, resulting in the impairment of their related functions in hemostasis and pigmentation [56].

#### 2.4.2. Clinical Manifestations

Some of its clinical manifestations are oculocutaneous albinism (OCA), meaning a reduction in retinal, skin and hair pigmentation, variable bleeding predisposition, visual acuity impairment, nystagmus, strabismus, as well as pulmonary fibrosis, granulomatous colitis, renal impairment, and immunodeficiency. The disease’s clinical course and prognosis are characterized by great heterogeneity. Ocular and skin findings commonly lead to early childhood diagnosis, with the skin and hair color ranging from pale white to olive and iris color tending to be blue, green, or brown. However, it should be taken into consideration that sometimes OCA is not easy to recognize, and suspicion arises only after comparing unaffected family members. Bleeding manifestations of HPS also vary and include excessive bruising, epistaxis, gingival bleeding, or other forms of mucosal bleeding, surgical or dental bleeding, menorrhagia and postpartum hemorrhage. Restrictive lung disease typically becomes clinically apparent in the early 30s and granulomatous colitis is severe in about 15% of affected individuals [10,57]. 

#### 2.4.3. Epidemiology of HMB

Hermansky–Pudlak syndrome is a rare disease and therefore our knowledge is limited. A systematic review of case reports and case series was conducted in 2020 by Deborah Obeng-Tuudah et al. about HPS and its obstetric and gynecological complications. According to its findings, HMB is the most common bleeding manifestation in women affected with the syndrome, reported in 53% (8/15) [55]. It is important to note that HPS can sometimes remain undiagnosed until an episode of HMB, due to its heterogeneity in clinical presentation, as indicated by cases in the literature. Therefore, it is of the utmost importance to consider it in women with HMB, especially if more common hematologic diseases have been excluded and clinical features, such as OCA, are present [58,59].

#### 2.4.4. Diagnosis

Suspicion of a delta storage pool disorder is based on the patient’s general features (such as OCA), family history, and a suggestive bleeding phenotype. Initial laboratory testing includes the bleeding time that is usually prolonged and a FBC that is within normal limits as far as the platelet count is concerned. After more common causes have been excluded, platelet aggregometry testing can be performed, which is usually abnormal in HPS. Typically, this consists of decreased aggregation response to arachidonic acid, collagen, and thrombin, but not ristocetin, as well as a deficient second-wave aggregation in response to epinephrine or ADP. Platelet light transmission electron microscopy provides a definitive diagnostic feature of delta storage pool disorders, a significant deficiency in platelet dense granules. As a final step of the diagnostic work-up, molecular genetic analysis allows specific diagnosis of the subtype of Hermansky–Pudlak syndrome, which is important for better management [56].

#### 2.4.5. Treatment Options for HMB

HPS is a rare disease and therefore there is no standard treatment for patients affected with HMB. Reports of HMB in HPS patients and their therapeutic interventions are scarce. In the following tables (Table 5 and Table 6) we have gathered eight cases from the literature and their respective therapies. The LNG-IUS has also been used with success in one patient with HPS [60].

### 2.5. Immune Thrombocytopenia

Immune thrombocytopenia, formerly known as Idiopathic thrombocytopenic purpura (ITP), is an autoimmune disorder characterized by reduced platelet counts (<100 × 10^9^/L) and increased bleeding risk in the absence of other causes associated with thrombocytopenia [62]. The overall prevalence of ITP in the general population is estimated to be 2 to 5 per 100,000 people [63], while in women of childbearing age, the estimated prevalence is 24.5 per million [64]. Young adults and especially women in the third and fourth decade of their life are the population group most frequently affected, with an overall female to male ratio of 2 to 1 [65].

#### 2.5.1. Pathophysiology

The pathogenesis of ITP includes the formation of autoantibodies against antigens expressed on platelet surface molecules. Platelets coated with IgG autoantibodies are rapidly cleared through binding Fcg receptors expressed by tissue macrophages, mainly in the spleen and the liver. In most patients, this leads to a compensatory increase in platelet production. In others, however, this compensation fails, due to either destruction of antibody-coated platelets by macrophages within the bone marrow or inhibition of megakaryocyte production. The level of thrombopoietin is not increased, reflecting the presence of the normal megakaryocyte mass. Antibodies that react with the glycoprotein IIb/IIIa complex, glycoproteins Ib/IX, Ia/IIa, IV, and V and diverse other platelet determinants have been identified, and it is typical for a patient with ITP to have antibodies against multiple antigens. The destruction of platelets by antigen-presenting cells leads to the creation of neoantigens, which subsequently drives the production of new autoantibodies in a vicious cycle, leading to further platelet destruction, sufficient to cause thrombocytopenia [66].

#### 2.5.2. Clinical Manifestations

While ITP in childhood is usually an acute, self-limiting condition (the thrombocytopenia is transient and recovers spontaneously despite an initially severe presentation), ITP is more often a chronic disease in adults with an insidious onset requiring multiple therapeutic approaches [65]. Bleeding symptoms, such as petechiae, unexplained ecchymoses, epistaxis, prolonged bleeding, and hematomas, are common manifestations in patients with ITP. Paradoxically, ITP patients also have increased risk of thromboembolic events [67]. Important symptoms for the patients, which are often overlooked, are those affecting their health-related quality of life, mainly fatigue (up to 45%), anxiety or depression (29%), and headache (16%) [67,68]. According to the World Impact Survey (iWISh), the most common patient-reported symptoms of ITP—by patients from 13 countries around the world—were petechiae (64%, diagnosis; 31%, survey completion) and bruising of unknown origin (65%, diagnosis; 30%, survey completion). The other two symptoms that concerned patients the most, were anxiety (34%, diagnosis; 32%, survey completion) and fatigue. Only 6% of patients were asymptomatic upon diagnosis and 13% upon completion of the survey accordingly. In the same survey, heavy menstrual bleeding was experienced by 439 out of 975 women at ITP diagnosis and 161 at survey completion, of whom 83% (n = 364/439) and 62% (n = 100/161) accordingly, rated it as one of their most severe symptoms. Furthermore, HMB, along with hematuria, rectal bleeding and prolonged bleeding episodes was one of the symptoms that physicians considered to have a major negative impact on ITP patients’ quality of life [62].

#### 2.5.3. Epidemiology

In a 2022 cohort study and review of the literature by van Dijk et al., the frequency of HMB among women with ITP ranged from 6% to 55% among the eight included studies, with the wide interval probably reflecting-other than differences in the studied population-also the different assessment methods used and the varying timing in the assessment of the patients; at diagnosis vs. during the course of disease [69].

#### 2.5.4. Diagnosis

Despite our knowledge of the pathophysiology of ITP, the diagnosis is still one of exclusion. Usually, the only abnormal lab finding is a low platelet count, while PT and aPTT are most often within the normal limits [63].

#### 2.5.5. Treatment Options

Explicit guidelines for the management of adult patients with immune thrombocytopenia have been published by the American Society of Hematology in 2019 [63]. In adults with newly diagnosed ITP who are asymptomatic or have minor mucocutaneous bleeding, the ASH guideline panel recommends the use of corticosteroids rather than observation for patients with a platelet count of <30 × 10^9^/L and observation for those with a count >30 × 10^9^/L. It should be noted that in the studies assessed by the expert panel there were none directly comparing corticosteroid use to observation in this patient population. The experts suggest that in those who are eligible for corticosteroid therapy, a short course (≤6 weeks) of oral prednizone or dexamethazone should be administered. In adults with ITP for ≥3 months who are corticosteroid-dependent or unresponsive to corticosteroids the panel recommends, by order of preference, a Thrombopoietin receptor agonist-specifically either eltrombopag or romiplostim-rituximab or splenectomy. However, it is mentioned that when the choice is made, several factors should be taken into account, including “duration of ITP, frequency of bleeding episodes requiring hospitalization or rescue medication, comorbidities, age of the patient, medication adherence, medical and social support networks, patient values and preferences, cost, and availability”. Furthermore, it should be noted that all the above-mentioned recommendations are based on evidence, which was evaluated as low to very low certainty by the panel [63,64].

#### 2.5.6. Treatment Options for HMB

The 2019 ASH Guidelines refer to the general management of all patients with ITP. However, for women with episodes of heavy menstrual bleeding, symptom-specific strategies, common to the disorders mentioned previously, may be needed. Available therapeutic options for HMB are hormonal therapy, endometrial ablation and hysterectomy. However, some of these treatments impair fertility permanently, thus, for women with ITP who wish to retain fertility, hormonal therapies, such as oral contraceptive pills (OCP) or intrauterine devices (IUD), combined with antifibrinolytic therapy are preferably used. These management options may successfully manage HMB [69,70,71]. In a cohort study, van Dijk et al. included 37 women with HMB and ITP, assessed by three prespecified criteria, specifically, PBAC score, clinical menstrual problems, and ITP-Bleeding Assessment Tool score. They also reported the management technique that each of them used. The frequencies of the techniques employed by the women in the study are summarized in Table 7.

Among the management techniques, LNG-IUD showed a benefit in reducing menstrual bleeding and improving quality of life assessed by the MMAS score; however, it is important to note that the study population is small, and the results cannot be safely generalized.

### 2.6. Joint Hypermobility

#### 2.6.1. Definitions and Clinical Manifestations

Joint hypermobility (JH) is not a diagnosis, but a descriptor that defines an excess in the range of motion in one or more joints considering age-, sex-, and race-specific mobility. Generalized joint hypermobility (GJH) is defined as the simultaneous presence of JH in the axial skeleton and the four limbs [72]. The Beighton score has been proposed as an objective measure of hypermobility and it is a nine-point assessment tool with points accumulated as follows: (a) ability to place palms flat on the floor while flexing forward with knees extended, (b) ability to hyperextend the elbow beyond 10°, (c) ability to hyperextend knee beyond 10°, (d) ability to bend thumb backward and touch the forearm, and (e) ability to bend little finger backward beyond 90° (Table 8). GJH is generally indicated by a score of ≥4/9 in adults, although there is not sufficient evidence-based justification for use of this cut-off value [72,73]. Alternatively, it has been proposed that the Beighton score should be considered positive (a) ≥6/9 in prepubertal children and adolescents, (b) ≥5/9 from puberty to 50 years of age and (c) ≥4/9 in people older than 50 years. This way the cut-off is considered more age-specific and thus aims in avoiding over-diagnosis in children, since joint range of motion decreases with age [11]. 

GJH can be symptomatic and a part of certain syndromes although it is also present in the general population in an asymptomatic form. Joint hypermobility syndrome (JHS) was originally conceived and described as a connective tissue disorder combining GJH and one or more systemic symptomatic complications, making it a part of a spectrum of diseases that include Ehlers–Danlos syndromes [73]. This syndrome showed phenotypic overlap with other heritable disorders of connective tissue (HDCT) [74]. Potential well documented morbidities associated with JHS, included arthralgias, gastrointestinal disorders, developmental retardation, as well as bleeding and bruising tendency [73]. The Brighton criteria were used in the past as a way of identifying the syndrome. They consisted of major and minor criteria and incorporated the Beighton score.

However, the 2017 International Classification of the Ehlers-Danlos syndromes (EDS) replaced previous terms for symptomatic and syndromic joint hypermobility (or joint hypermobility syndrome) with hypermobile EDS (hEDS). It also introduced the term hypermobility spectrum disorders (HSD) for the great range of patients facing joint hypermobility with symptomatic systemic complications, but who do not meet the diagnostic criteria for hypermobile EDS. The 2017 Diagnostic Criteria are applied to diagnose this spectrum of conditions, as indicated in Table 9 and Table 10 [11]. Ehlers–Danlos syndromes comprise a clinically heterogeneous group of 13 diseases characterized by abnormalities in one of several types of extracellular matrix proteins such as collagen [75]. Hypermobile EDS, the most common type of EDS, is a condition inherited in an autosomal dominant pattern, but there is to date no known genetic mutation to help with diagnosis, in contrast to all other forms of EDS where a genetic component has been identified. Therefore, the diagnosis of hEDS is based on clinical picture, and there are no laboratory tests to contribute to it. Likewise, HSD is also a solely clinical diagnosis as mentioned above. HSD are a group of conditions that resemble in many ways hEDS but do not meet its diagnostic criteria [11,75].

The clinical pictures of hypermobile EDS and HSD are variable. Common manifestations include joint hypermobility, chronic joint pains, recurrent dislocations, subluxations and skin hyperextensibility. However, it is of utmost importance to note that EDS as well as HSD include a wide range of symptoms affecting almost every system, in multiple possible combinations. Some of their extra-articular manifestations include anxiety disorders, depression, chronic fatigue, orthostatic intolerance, functional gastrointestinal disorders, pelvic and bladder dysfunction, pelvic organ prolapse, as well as easy bruising, excessive bleeding in surgeries, and heavy menstrual bleeding [11,75].

#### 2.6.2. Epidemiology

Joint hypermobility as defined by the Beighton score is not rare at all, but the exact prevalence depends on the BS score cut-off value and the population studied. It has been recorded in up to 38.5% of young women and 25.4% of young men when a Beighton score greater than 4 is considered positive [78]. More recent studies on school-aged children have estimated it to be closer to 5.7% when the cut-off is 6 and up to 19.2% when the cut-off value is 4 [79]. It is important to note that in most of those studies, when men and women are recorded separately, there seems to be a significant female prevalence against men [78]. The percentage of people with GJH that end up being diagnosed with EDS or HSD is still unknown. Symptomatic GJH, as a combination of GJH and other systemic symptoms, has been estimated to be present in up to 2 to 3 people per 1000 individuals [11,75]. Some of the best estimates to date, about the combined prevalence of EDS and HSD, estimate a proportion of about 2 patients for every 1000 individuals in the general population [80]. The most common EDS subtype, hEDS, has an estimated prevalence of 1–3 out of 10,000 individuals. In this subcategory, still there seems to be a significant female prevalence with a 9/1 female to male ratio [75].

#### 2.6.3. Epidemiology of HMB

To date, there is very little published research dedicated to the gynecologic symptoms of females with GJH, and this is an area in need of further investigation. However, available data do suggest abnormal uterine bleeding, and other gynecological symptoms are common manifestations of these disorders. In a cohort study including 386 women with hypermobility type Ehlers-Danlos syndrome, 76% of them had a history of menorrhagia/HMB [81]. In another study, including 82 post pubertal women with hEDS, 53,7% had AUB [82]. Additionally, HMB associated with hypermobility spectrum disorders not only seems to be common but also quite often to negatively impact the quality of life or end up being life-threatening. In a retrospective review of 30 adolescents with GJH and AUB, 80% described their menstrual flow as “heavy” or “very heavy” according to a Menstrual Impact Questionnaire. HMB was severe enough to negatively impact school performance and extracurricular activities in as many as 60% of subjects. In addition, 43% of patients were anemic. The same study highlights that, a significant delay occurred between menarche and referral to specialty care [73]. Finally, in a cohort of 52 EDS patients, 7 of 52 (14%) experienced HMB that was life-threatening or required surgery [83].

#### 2.6.4. Pathophysiology

The causes of the hemostatic problems in patients with EDS and HSD are yet to be clarified, but have long been hypothesized to be primary hemostatic disorders. Collagen structure is altered in many EDS patients, although genes responsible for hEDS have not been discovered yet. Deformed collagen leads to weakened vessel walls, as well as, defective interaction with platelets and Von Willebrand factor, thus hindering platelet plug formation. hEDS has also been related to mast cell disorders which independently can lead to a bleeding tendency [77]. Interestingly, EDS has also been linked to platelet function abnormalities. Specifically, the more platelet abnormalities are detected, the greater the bleeding risk [84].

#### 2.6.5. Treatment Options for HMB

Research in the field of therapeutic interventions for the management of HMB in this disease category is scarce. Most therapeutic attempts follow the general recommendations of HMB treatment, although early referral of adolescents to specialist care is recommended, aiding towards a more targeted approach. In the same study mentioned above by Kendel et al. with the 30 patients with GJH and HMB, most of the patients, 68% (20/30), required escalation of their initial therapeutic approach and only 32% (10/30) achieved adequate control of their HMB with the initial treatment selections, indicating GJH as a risk factor of difficult management. The treatment options that eventually lead to control of HMB are indicated on Table 11 [73]. In another study of 26 EDS (25 hEDS and 1 vascular EDS) patients, of which 24 (92%) had menstrual complaints, 13 (50%) reported HMB although the most common was dysmenorrhea. Their menstrual problems were controlled in 9/24 (37,5%) with only one method, with the most common one being progesterone only pills in 44%. 15/24 (62,5%) required 2 or more escalations of therapy, with the most widely used option being DMPA in 73% and with most popular final choice being LNG-IUS in 27% [85]. The use of LNG-IUS has also been implicated with success in EDS patients with HMB in other studies too [86].

## 3. Conclusions

HMB is a common gynecological problem affecting women of reproductive age and compromising their quality of life. When HMB is diagnosed, the C category according to the PALM-COEIN system represents a major group of hematologic clinical entities that have to be included in the differential diagnosis. VWD is the most common disorder in the C category and the most studied disease of those, as a cause of HMB. When the basic hematologic workup has been completed, usually the attention is directed towards other causes outside the C category. However, primary hemostasis disorders can be a cause of HMB even without affecting PT, aPTT and sometimes with normal CBC. Under those circumstances, the clinician must suspect and further investigate the possibilities of hematologic diseases. This review aimed at summarizing the basic findings regarding primary hemostasis disorders and their association with HMB. It includes basic information regarding their epidemiology, pathophysiology, clinical picture, and treatment in a succinct way, in order to act as a reference guide for the clinician. It includes VWD, as well as other causes that could potentially be missed because of their rarity. It acts as a reminder that diagnostic workup can have more steps even when most common diseases have been excluded and emphasizes the importance of a thorough physical examination and a clinical acuity, as indicated by HSD and EDS, platelet disorders and ITP. A simple and easy to use clinical tool such as the Beighton score could be used by both primary care providers and gynecology specialists, in order to pick out those cases that could otherwise be easily missed due to their perceived rarity and their negative laboratory workup. After that point, a specialist hematologist consultation is of great importance in order to reach an accurate diagnosis. This review also aims to provide a disease-specific summary of evidence about each clinical entity mentioned, in order to guide towards a more targeted approach. As a literature and not a systematic review it provides a non-exhaustive view of the topic based on the published literature, and it should not be used as a sole resource to guide diagnostic decisions or therapeutic interventions, but rather as an informative source and a reference for further reading. Further research is needed in order to clarify a possible connection between the remaining rare primary hemostasis disorders and HMB, as well as to gather more solid data about the diseases mentioned and their clinical and therapeutic approach.

## Figures and Tables

**Table 1:** Reported prevalence of HMB or menorrhagia among women with VWD.

	Study Population	Prevalence of HMB
Ragni et al., 2016 [23]	1321 women with VWD from 20 US Hemophilia Treatment Centers, 18–45 years old, seen during 2012–2014.	Heavy menstrual bleeding reported by 816 (61.8%) women with VWD.
Sanders et al., 2014 [24]	664 adults with Von Willebrand disease, as compared with 500 healthy persons, in the Willebrand in the Netherlands (WiN) study.	More than 80% of women with VWD experienced menorrhagia.
de Wee et al., 2011 [25]	423 women aged ≥ 16 years old with moderate and severe VWD in the Netherlands.	Menorrhagia, defined as occurrence of ≥2 menorrhagia symptoms, was reported by 81%.
Kadir et al., 1998 [26]	150 women referred for investigation of menorrhagia whose pelvis was normal on clinical examination and who had an estimated menstrual blood loss of more than 80 mL.	13% VWD prevalence. Menorrhagia since menarche 65% of 20 women with Von Willebrand disease compared with 8,9% of 123 women without a bleeding disorder.
Woods et al., 2001 [27]	1885 patients of all ages with VWD–1142 females—from a reference center in Argentina.	47% of women more than 13 years old.

**Table 2:** Comparative studies of therapeutic interventions for the management of heavy menstrual bleeding.

Therapeutic Interventions	Source of Evidence	Study Population	Results
Desmopressin vs. TxA	1 Randomized clinical trial [31]	116 women 18–50 years old with HMB and coagulation disorder.	(1) Average estimated decrease in PBAC * score from baseline: −64·1 (95% CI = −88·0, −40·3) for IN-DDAVP and −105·7 (95% CI = −130·5, −81·0) for TxA. (2) Normalization of menstrual bleeding (PBAC < 100): 22% for IN-DDAVP vs. 33% for TxA.
Desmopressin vs. hormonal therapy	1 Observational study [32]	36 adolescent females 9–18 years old with HMB and VWD.	Alleviation of symptoms (median follow-up 30 months): 77.1% DDAVP vs. 85.7% hormonal therapy RR 0.90; 95% CI, 0.66–1.23).
TxA vs. recombinant VWF	1 Randomized cross-over clinical trial [33]	36 women 13–45 years old with mild or moderate VWF and HMB.	Median PBAC score significantly lower after two cycles with tranexamic acid vs. recombinant VWF (146 (95% CI 117–199) vs. 213 (152–298); adjusted mean treatment difference 46 (95% CI 2–90); *p* = 0·039). (median follow-up 23.97 weeks).

* Pictorial Blood Assessment Chart (PBAC) score: PBAC is a visual scoring system, widely used to estimate menstrual blood loss, using a graded series of soiled tampons and/or towels. In all three studies, women with a score >100 were considered to have heavy menstrual bleeding.

**Table 3:** Treatment in 18 patients with GT and HMB [41].

Number of Patients	Treatment Method
16/18 (88.9%)	Hormonal Methods
15/18 (83.3%)	Blood products
9/18 (50%)	Antifibrinolytics
4/18 (22.2%)	rFVIIa
4/18 (22.2%)	Surgical interventions
3/18 (16.7%)	Iron Supplementation

**Table 4:** Treatment in 14 patients with BSS and HMB [41].

Number of Patients	Treatment Method
13/14 (92.9%)	Blood products
11/14 (78.6%)	Hormonal methods
9/14 (64.3%)	Iron supplementation
8/14 (57.1%)	Antifibrinolytics
3/14 (21.4%)	rFVIIa
3/14 (21.4%)	Surgery
2/14 (14.3%)	DDAVP
3/14 (21.4%)	Other (uterotonic agents, steroids and IVIG)

**Table 5:** Case reports on treatment of HMB in women with HPS.

Publication	Patient	Treatment
J Lohse et al. [58]	13-year-old with 14 days HMB at menarche that led to diagnosis of HPS	8 PRBCs, 1 FFP and Norethisterone could not control the bleeding
		After suspicion of the diagnosis 5 doses of IV desmopressin and IV tranexamic acid were also not effective. Then, rFVIIa was used and bleeding was controlled. She continued during her periods with progesterone and TXA for further prevention in the long run.
Ray A et al. [59]	42-year-old with 7-month history of HMB that led to diagnosis of HPS	PRBCs (unknown number), Oral contraceptives, TxA and Desmopressin that controlled her bleeding. She continued with TxA and desmopressin during her periods.

**Table 6:** Patient case series by Joel Rivera-Concepción et al. [61].

Number of Patients	Treatment Method
4/6 (66.7%)	Hormonal therapy
2/6 (33.3%)	Aminocaproic acid
2/6 (33.3%)	Desmopressin
2/6 (33.3%)	Surgical intervention (1 hysterectomy and I endometrial ablation)
1/6 (16.7%)	Platelet transfusion
1/6 (16.7%)	Iron supplementation

**Table 7:** Frequency of management method use by women with HMB and ITP based on the results of the study by van Dijk et al. [69].

Management Method	Number of Patients
OCP monotherapy	7 (19%)
LNG-IUD monotherapy	10 (27%)
Hormonal therapy, other	2 (5%)
OCP + TxA	2 (5%)
LNG-ICP + TxA	1 (3%)
TxA + IVIG	1 (3%)

**Table 8:** The Beighton score.

Passive Hyperextension of the Fifth Metacarpophalangeal Joint beyond 90°	1 Point for Each Side of the Body (Left, Right)
Passive apposition of the thumb to the forearm	1 point for each side
Hyperextension of the elbow beyond 10°	1 point for each side
Hyperextension of the knee beyond 10°	1 point for each side
Placing of palms flat on the floor while flexing forward the spine with knees extended	1 point
	Sum/9

**Table 9:** Hypermobility spectrum classification.

Hypermobility Type Diagnosis	Beighton Score	Musculoskeletal Manifestations *	
Hypermobile Ehlers–Danlos Syndrome	Positive	Possible	Meets diagnostic criteria for hEDS
Hypermobility Spectrum Disorders (HSD)			
Generalized HSD	Positive	Present	Does not meet criteria for hEDS
Peripheral HSD	Usually negative	Present	JH limited to hands/feet
Localized HSD	Negative	Present	JH limited to certain joints or body parts
Historical HSD	Negative	Present	Reported historic presence of JH
Asymptomatic non-Syndromic JH			
Asymptomatic generalized JH	Positive	Absent	
Asymptomatic peripheral JH	Usually negative	Absent	JH limited to hands/feet
Asymptomatic localized JH	Negative	Absent	JH limited to certain joints or body part (usually less than 5 joints)

Based on [11,75], * Musculoskeletal manifestations refer to: (a) chronic pain, (b) disturbed proprioception, (c) musculoskeletal or/and soft tissue trauma, and (d) other musculoskeletal conditions (flexible flat feet, scoliosis, kyphosis, lordosis, misaligned bones in the elbow and big toe).

**Table 10:** Simplified diagnostic criteria of hEDS.

Diagnosis of hEDS Requires Simultaneous Presence of Criteria 1, 2 and 3
Criterion 1: Generalized joint hypermobility (GJH) as indicated by the Beighton score (a) ≥4/9 for those >50 years of age (b) ≥5/9 for pubertal men and women up to the age of 50 (c) ≥6/9 for pre-pubertal children and adolescents
Criterion 2: 2 of 3 must be met (a) ≥5/12 systemic findings in skin, heart, etc. (b) Family history of hEDS (c) Joint pain or instability
Criterion 3: Absence of other causes of symptoms (a) Absence of other types of EDS (b) Exclusion of other connective tissue disorders (c) Exclusion of other causes of joint hypermobility

Based on [76,77].

**Table 11:** Treatment in 30 patients with HMB and GJH [73].

Number of Patients	Treatment Method
19/30 (63%)	Long-term oral hormonal therapy
3/30 (10%)	Oral hormonal therapy + intrauterine device (IUD)
3/30 (10%)	DMPA (Depo Medroxyprogesterone Acetate)
2/30 (6.6%)	IUD
2/30 (6.6%)	Tranexamic acid
1/30 (3.3%)	Oral hormonal therapy +IUD +leuprolide acetate

## Data Availability

Data used in this study are presented within the manuscript.
